# Increasing cotton lint yield and water use efficiency for subsurface drip irrigation without mulching

**DOI:** 10.3389/fpls.2024.1433719

**Published:** 2024-07-25

**Authors:** Nan-nan Li, Jun-hong Li, Xiao-juan Shi, Feng Shi, Yu Tian, Jun Wang, Xian-zhe Hao, Hong-hai Luo, Zhan-biao Wang

**Affiliations:** ^1^ Key Laboratory of Oasis Eco-Agriculture, Xinjiang Production and Construction Group, Shihezi University, Shihezi, Xinjiang, China; ^2^ Institute of Western Agriculture, The Chinese Academy of Agricultural Sciences, Changji, China; ^3^ Soil and Water Research Institute, Xinjiang Academy Agricultural and Reclamation Science, Shihezi, China

**Keywords:** subsurface drip irrigation, without mulching model, soil hydrothermal environment, water use efficiency, photosynthesis

## Abstract

**Introduction:**

Planting without mulching can eliminate the residual film pollution caused by the long-term use of plastic film covers, but it will increase soil moisture evaporation and heat loss and severely reduce water use efficiency and cotton productivity in cotton (*Gossypium hirsutum* L.) fields in arid regions. It is unclear whether the advantages of subsurface drip irrigation and nighttime irrigation can be leveraged to reduce the amount of irrigation applied in fields, improve the soil and leaf hydrothermal environments, and increase the synchronicity of yield and water use efficiency (WUE).

**Methods:**

Therefore, in a two-year field experiment (2019-2020), cotton was grown under different irrigation treatments (I5, 3753 m^3^ ha^-1^; I4, 3477 m^3^ ha^-1^; I3, 3201 m^3^ ha^-1^; I2, 2925 m^3^ ha^-1^; and I1, 2649 m^3^ ha^-1^). The soil volumetric moisture content, soil temperature, leaf relative water content (RWC), daily changes in gas exchange parameters, lint yield, and WUE were evaluated.

**Results and discussion:**

The results showed that reducing irrigation can reduce the soil volumetric moisture content (0-40 cm soil layer), increase the soil temperature and soil temperature conductivity, and increase the leaf temperature, intercellular carbon dioxide concentration (Ci), and WUE; however, reducing irrigation is not conducive to increasing the leaf RWC, net photosynthetic rate (Pn), stomatal conductance (Gs), or transpiration rate (Tr). There was no significant difference in WUE between the I3 and I4 treatments from 8:00 to 20:00, but the lint yield in these treatments increased by 2.8-12.2% compared to that in the I5 treatment, with no significant difference between the I3 and I4 treatments. In addition, a related analysis revealed that the positive effects of the leaf hydrothermal environment on the Pn and soil temperature on the WUE occurs during the same period (10:00-16:00). Overall, an irrigation amount of 3201-3477 m^3^ ha^-1^ applied with a subsurface nighttime irrigation system without mulching can enhance the soil moisture content and soil temperature, maintain a high photosynthetic capacity, and increase the lint yield and WUE. These results revealed that the negative impacts of plastic film contamination in arid areas can be alleviated.

## Introduction

Cotton (*Gossypium hirsutum* L.) is an important heat-loving crop that is a source of fiber and oil worldwide (Wu et al., 2018). Low temperatures constitute a natural disaster that affects the growth and distribution of this crop. At low temperatures, the active oxygen species in plants becomes unbalanced, photosynthesis is inhibited, growth and development are delayed, and yield and quality decrease (Barrero Gil and Salinas, 2013; Wu et al., 2018). The promotion of plastic film mulching planting technology has effectively eliminated these effects, as it has multiple advantages, such as increasing temperature, conserving soil moisture, improving crop yield, early harvesting, and improving water use efficiency (WUE) (Hu et al., 2020; Yang et al., 2020). However, the increase in the use of plastic film mulching has led to plastic film pollution and has caused incalculable losses in terms of soil quality and crop production, for example, by hindering water and fertilizer transfer, reducing fiber quality, endangering normal crop growth and development, and causing crop yield reduction; thus, this has become a complicated issue in agricultural development (Li et al., 2014; Yang et al., 2021; Wen et al., 2023). Due to limitations associated with residual film recycling technology and the influences of regional production costs, the ecological environment, climate conditions, and soil properties, the promotion and production of plastic film recycling technology and plastic film replacements, such as straw covering technology and biodegradable film, face large obstacles (Scarascia-Mugnozza et al., 2006; Wang et al., 2019; Ding et al., 2021; Shi et al., 2023). The ‘without mulching’ planting model eliminates pollution at the source from residual film on raw cotton, protects the ecological environment of cotton fields, and might be an alternative that can be gradually implemented in dryland agriculture (Li et al., 2023; Wang et al., 2023).

Photosynthesis is an important pathway for studying the impacts of environmental factors on plant growth via metabolism, and diurnal changes in photosynthesis can intuitively reflect the trend of changes between plant photosynthetic capacity and the environment (Zhang et al., 2008). Compared with the mainstream film mulching planting model, the without mulching planting model has a lower soil temperature during the early growth stage of plants, which is not conducive to crop growth and development, especially photosynthesis (Hirotse et al., 2005). It is generally believed that low temperatures hinder plant chlorophyll synthesis, cause stomata to close, and lead to reductions in CO_2_ absorption and the photosynthetic rate, which impedes the transport of photoassimilates to reproductive organs (Jia et al., 2019). Moreover, different soil moisture contents can greatly affect the gas exchange parameters of cotton leaves, reducing yield (Miao et al., 2023). Therefore, it is necessary to study the relationships between the photosynthetic capacity of cotton and environmental factors in the absence of mulching.

China’s cotton production accounts for 25.4% of global production, and Xinjiang has the largest irrigated cotton production area in China. In 2021, cotton production in Xinjiang accounted for 89.5% of the country’s total cotton production (Shi et al., 2022). However, water scarcity has greatly affected the development of cotton production in the region, where the annual rainfall is 50-250 mm, which is only 28.1% of the national average (Zong et al., 2021). Subsurface drip irrigation can minimize evaporation loss and enable dry upper soil to capture seasonal rainfall, greatly improving irrigation water efficiency and yield (Aydinsakir et al., 2021a). Studies have shown that subsurface drip irrigation can optimize the distribution of water among soil layers, reduce surface evaporation, eliminate deep seepage, alleviate environmental pollution caused by excessive irrigation, maintain high surface temperatures, increase the net photosynthetic rate of crops and prevent a decrease in yield without film coverage (Çolak et al., 2017; He, 2021; Aydinsakir et al., 2021b). Research has shown that drip irrigation at an optimal depth of 15 cm underground can increase the photosynthetic capacity of cotton plants and ensure the maximum economic coefficient (Duan et al., 2022). In addition, nighttime irrigation can reduce the stimulating effect of irrigation water temperature on crop roots, increase daytime photosynthesis, delay leaf aging, and improve single boll weight (Wang et al., 2022).

Hence, the main purpose of this paper was to utilize the advantages of subsurface drip irrigation and nighttime irrigation, optimize the irrigation amount, create a favorable soil and leaf hydrothermal environment to enhance the photosynthetic capacity, achieve lint yield improvement, and reduce plastic film pollution in cotton fields. Specifically, this focused on (i) exploring the effects of different irrigation amounts on diurnal changes in soil temperature, RWC, gas exchange parameters, yield, and WUE in a nighttime deep drip irrigation planting system without mulching under limited water availability; (ii) proposing a plan to maximize yield and WUE in the system; and (iii) clarifying the feasibility of using a subsurface nighttime irrigation system without mulching to replace drip irrigation with mulching in cotton cultivation to reduce the harmful effects of residual film pollution.

## Materials and methods

### Experimental site and cultivar

The experiment was conducted from 2019 to 2020 at the Shihezi Experimental Station for Crop Water Use of the Ministry of Agriculture (45°38′ N, 86°09′ E), Xinjiang. The soil texture was largely sandy loam, the pH and electrical conductivity of the 0-20 cm soil layer at the experimental site were 7.86 and 567 μS cm^-1^, respectively, and the contents of total N, available potassium, available phosphorus, and organic matter were 1.3 g kg^-1^, 174 mg kg^-1^, 29 mg kg^-1^, and 23 g kg^-1^, respectively. The cotton variety planted was Xinluzao 74 (120 d growth period). The average temperature and precipitation during the growth periods from April to October in 2019 and 2020 are shown in **Table 1**.

**Table 1:** Precipitation distribution, average daily maximum temperature, average temperature, average daily minimum temperature, and changes in growth period from April to October 2019 to 2020.

Month	2019				2020			
	Average temperature (°C)	Average daily maximum temperature (°C)	Average daily minimum temperature (°C)	Rainfall (mm)	Average temperature (°C)	Average daily maximum temperature (°C)	Average daily minimum temperature (°C)	Rainfall (mm)
Apr.	15.2	22.2	9.1	29.8	17.5	25.9	9.8	0.3
May	17.7	25.4	11.4	67.6	22.5	29.9	15.2	2.9
Jun	23.8	31.2	16.9	19.0	23.5	30.7	16.2	27.3
Jul.	26.1	33.7	18.9	25.3	25.4	32.7	18.3	13.6
Aug.	24.9	33.2	17.5	10.2	24.3	32.3	16.7	10.9
Sep.	19.1	27.9	12.0	25.2	17.2	24.9	9.9	1.2
Oct.	9.4	17.7	3.3	12.6	8.4	16.0	1.7	8.6

### Experimental design and management

**Figure 1:**
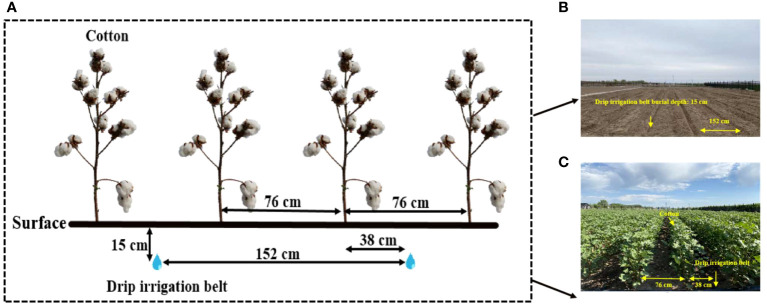
Layout **(A)** and pictures **(B, C)** of the field experiment.

The conventional 3753 m^3^ ha^-1^ irrigation amount in mulched cotton fields was used as a control (Gao et al., 2019), and on this basis, the irrigation amount was reduced in an equal gradient to produce five deep drip irrigation treatments, I5, 3753 m^3^ ha^-1^; I4, 3477 m^3^ ha^-1^; I3, 3201 m^3^ ha^-1^; I2, 2925 m^3^ ha^-1^; and I1, 2649 m^3^ ha^-1^ (**Table 2**). Four repetitions were performed per treatment, and we used a randomized complete block design. Importantly, no plastic film was used to cover any of the treatments in this experiment. The plot area was 72.96 m^2^ (4.56 m×16.0 m). Every two rows of cotton were equipped with 1 drip irrigation pipe (Netafim, Israel) **Figure 1**. The burial depth, inner diameter, thickness, and distance between built-in emitters were 15 cm, 16 mm, 1 mm, and 30 cm, respectively, and the drip flow rates were 1.0 L h^-1^ (2019) and 2.0 L h^-1^ (2020). The irrigation event was completed between 19:00 p.m. and 10:00 a.m., and the treatment was controlled by a water meter and an on/off ball valve. The seeds were sown on May 10, 2019, and May 5, 2020, and the seedling densities during the cotyledon spreading periods were 192,000 plants ha^-1^ and 232,500 plants ha^-1^, respectively. The seeds were sown at a distance of 38 cm from the drip irrigation pipe, with a sowing depth of 3.0~3.5 cm. After sowing, the seeds were irrigated with 300 m3 ha-1 water. Throughout the growth period, 525 kg ha^-1^ urea (46% N) and 150 kg ha^-1^ potassium dihydrogen phosphate (52% P_2_O_5_ and 34% K_2_O) were applied dropwise to the water. Weeds, insects and diseases were effectively managed by chemical spray when needed to prevent yield loss.

**Table 2:** Irrigation (m^3^ ha^−1^) amounts under the different treatments from 2019–2020.

Growth period	2019						Growth period	2020					
	Date	I_1_	I_2_	I_3_	I_4_	I_5_		Date	I_1_	I_2_	I_3_	I_4_	I_5_
Before emergence	5/10	300	300	300	300	300	Before emergence	5/5	300	300	300	300	300
Seedling	6/15	162	180	198	216	234	Seedling	6/22	180	210	240	270	300
	6/22	162	180	198	216	234	Squaring	7/2	240	270	300	330	360
	6/29	162	180	198	216	234		7/11	270	300	330	360	390
Squaring	7/1	189	210	231	252	273	Boll setting	7/19	314	345	377	408	440
	7/3	189	210	231	252	273		7/26	342	375	408	441	474
	7/5	189	210	231	252	273		8/3	314	345	377	408	440
	7/9	216	240	264	288	312		8/11	270	300	330	360	390
	7/15	216	240	264	288	312		8/20	240	270	300	330	360
Boll setting	7/21	246	270	294	318	342		8/30	180	210	240	270	300
	7/27	246	270	294	318	342							
	8/7	189	210	231	252	273							
	8/20	129	150	171	192	213							
	8/30	54	75	96	117	138							
Total		2649	2925	3201	3477	3753	Total		2649	2925	3201	3477	3753

### Soil volumetric moisture content

From 32 to 109 days after emergence (DAE), a portable soil humidity meter (HMSCAN-2 M, USA) was used to measure the volumetric moisture content at 10 cm intervals for the 0-80 cm soil layer every 7 days.

### Soil temperature and thermal conduction

At 55, 75 and 95 DAE, button temperature recorders (Maxim, DS1921G) were installed in the seeding lines to measure the soil temperature. The temperature collection depths were 5, 10, 20, 30 and 40 cm. The measurements were conducted from 0:00 to 24:00, and the collection interval was every 2 hours. The nighttime soil temperature data were collected from 0:00-10:00, and the daytime soil temperature data were collected from 10:00-22:00.

The thermal condution of each treatment was calculated (Equation 1). In the following equation, Grad T is the soil temperature gradient (°C m^-1^), and T and Z represent the temperature difference (°C) and depth difference (m) between the two layers, respectively. The temperature difference between the two layers is ΔT_1_=T_10_-T_5_, ΔT_2_=T_20_-T_10_, ΔT_3_=T_30_-T_20_, ΔT_4_=T_40_-T_30_, and the depth difference is ΔZ_1_ = 0.05_ m_, ΔZ_2_= ΔZ_3_=ΔZ_4_ = 0.10_ m_. T_5_, T_10_, T_20_, T_30_ and T_40_ represent the average daily ground temperatures of the 5, 10, 20, 30 and 40 cm soil layers, respectively.


(1) $$\text{Grad T}=\frac{\partial \text{T}}{\partial \text{Z}}\approx \left(\frac{{\text{ΔT}}_{1}}{{\text{ΔZ}}_{1}}+\frac{{\text{ΔT}}_{2}}{{\text{ΔZ}}_{2}}+\frac{{\text{ΔT}}_{3}}{{\text{ΔZ}}_{3}}+\frac{{\text{ΔT}}_{4}}{{\text{ΔZ}}_{4}}\right)$$

### Leaf relative water content

At 70, 90 and 110 DAE, the RWC of the third main stem leaf below the apex was measured. A punch was used to collect samples, avoiding leaf veins, and 5 circular slices were taken from each functional leaf to measure the fresh weight (FW). The extracted slices were immersed in distilled water for 24 hours to enable the tissue to fully absorb water and reach saturation, and their saturated fresh weight (SFW) was then determined. The material was then dried to constant weight at 80 °C, after which the dry weight (DW) was determined. The Equation 2 was used (Gao et al., 2021):


(2) $$\text{RWC\%}=\frac{\text{FW}-\text{DW}}{\text{SFW}-\text{DW}}\times \;100\;$$

### Gas exchange parameters

At 70, 90, and 110 DAE, the data were collected under clear and cloudless weather conditions. Using an LI-6800 portable photosynthesis measuring instrument (LI-COR, Inc., Lincoln, NE, USA), a standard leaf chamber equipped with red and blue light sources (2 cm×3 cm) was used, the flow rate was controlled at 500 μmol m^-2^ s^-1^, and the fan speed was 10,000 rpm. The net photosynthetic rate (Pn), transpiration rate (Tr), intercellular carbon dioxide concentration (Ci), stomatal conductance (Gs), and leaf temperature of the third main stem leaf below the apex were measured directly on the cotton plants every 2 hours from 8:00 to 20:00. Before recording three repeated measurements, each leaf was subjected to at least 20 minutes of equilibration.

### WUE and lint yield

WUE (μmol mmol^-1^) was expressed as the ratio of the Pn (μmol m^-2^ s^-1^) to the Tr (mmol m^-2^ s^-1^) (Liao et al., 2021); that is Equation 3:


(3) $$\text{WUE }=\frac{\text{Pn}}{\text{Tr}}$$

During the harvest period, representative sampling points (3 m × 1.52 m) were selected for each treatment, and all the bolls were harvested twice a year on October 15 and 25, 2019, and September 28 and October 8, 2020. The seed cotton yield was ginned to determine the lint yield. Each process was repeated 3 times to calculate the lint yield.

### Data analysis

Microsoft Office Excel 2019 (Microsoft, Redmond, USA) was used to organize and analyze the data, SPSS 25.0 (IBM, Armonk, USA) was used to conduct correlation analysis and Duncan’s multiple comparison test, and the significance level was set at 0.05 (P< 0.05). Origin 2021b (OriginLab, Northampton, USA) was used for plotting the data. The data are presented as the means ± SEs. In addition, Origin 2021b software was used to fit the quadratic polynomial regression of leaf temperature with daily progression, which is represented as y=ax^2^+bx+c, where y is the measured leaf temperature (°C); x is the time (hour of day); and a, b, and c are the fitting coefficients of the regression equation.

## Results

### Soil volumetric moisture content

**Figure 2:**
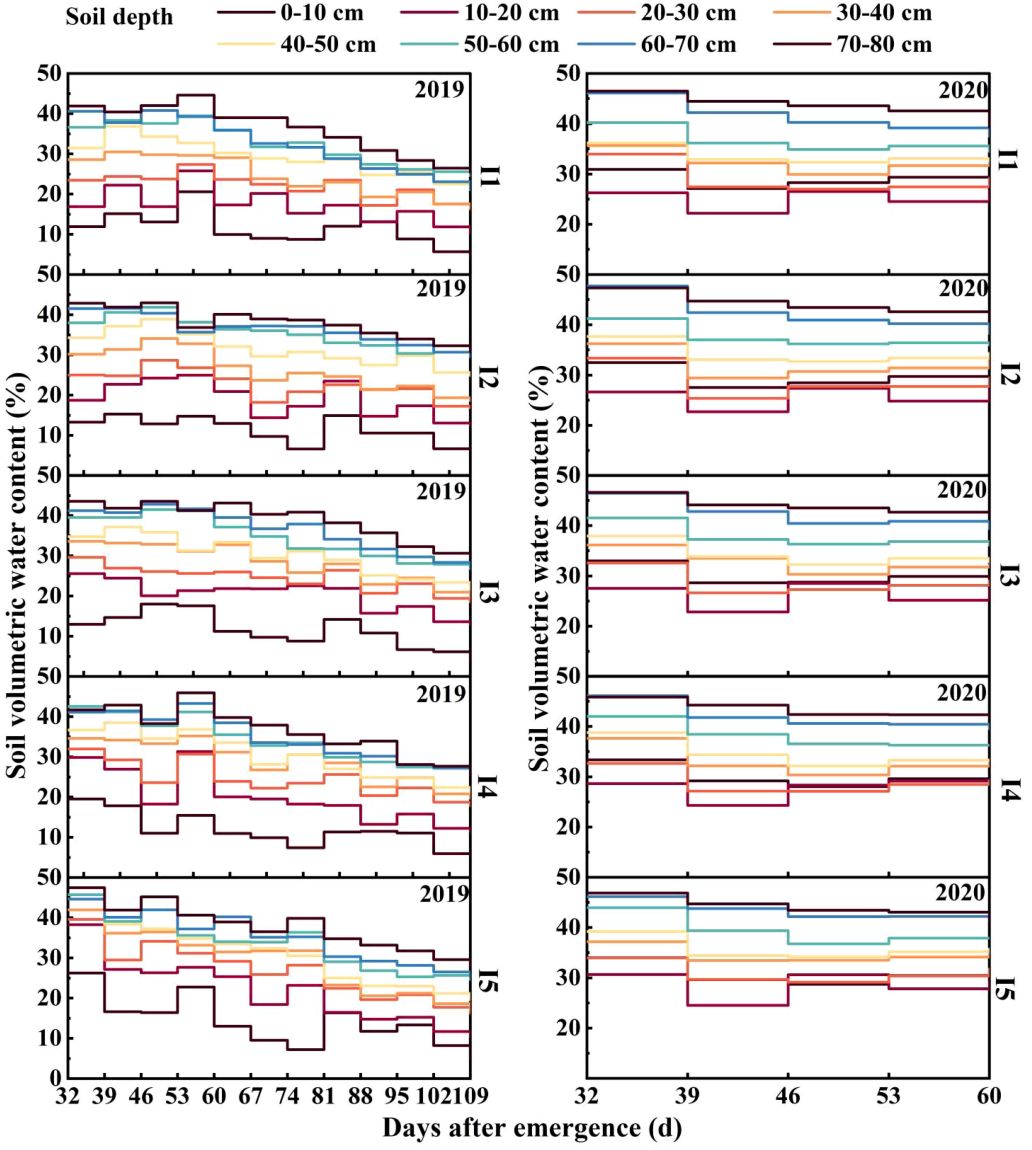
Effects of different irrigation amounts on soil volumetric moisture content in days after emergence in 2019 and 2020.

The average soil volumetric moisture content in the 0-80 cm soil layer increased with increasing drip irrigation amount, ranging from 32-46 DAE (**Figure 2**). On average, the soil volumetric moisture content in the 40-80 cm soil layer in the I1 treatment was 1.5%, 1.4%, and 3.4% lower than that in the I3, I4, and I5 treatments, respectively, during 2019-2020. From 46-60 DAE, the average two-year soil volumetric moisture contents of the 0-40 cm and 40-80 cm layers in the I5 treatment were 2.4-3.7% and 0.7-1.9% greater than those in the other treatments, respectively. In 2020, from 67-88 DAE, the soil moisture contents within the 0-20 cm and 30-40 cm soil layers in the I1 treatment were the lowest and were 0.1-2.1% and 0.8-4.0% lower than that in the other treatments, respectively. From 88-109 DAE, the soil moisture content in the 40-80 cm soil layer decreased by 21.2-35.0% compared to that from 0-88 DAE. The I3 and I4 treatments showed average increases of 3.1% and 1.9%, respectively, compared to the I1 treatment.

### Soil temperature

**Figure 3:**
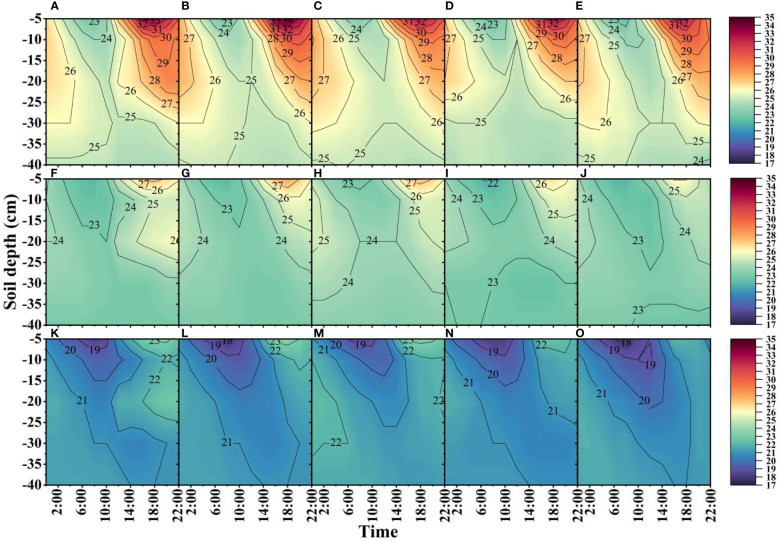
Effects of different irrigation amounts on the daily variation in soil temperature (°C). **(A–E)**, **(F–J)**, and **(K–O)** represent treatments I1-I5 at 55, 75, and 95 days after emergence in 2019, respectively. The contours represent the means of three repetitions.

**Figure 4:**
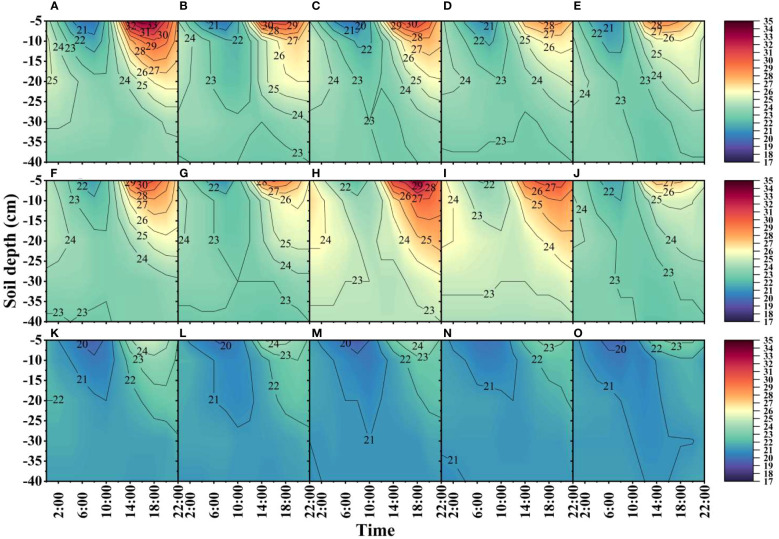
Effects of different irrigation amounts on the daily variation in soil temperature (°C). **(A–E)**, **(F–J)**, and **(K–O)** represent treatments I1~I5 at 55, 75, and 95 days after emergence in 2020, respectively. The contours represent the means of three repetitions.

From 55-95 DAE, the daily average soil temperature in the I1 treatment was 1.3-3.6% greater than that in the I4 treatment and 0.6-3.6% greater than that in the I5 treatment. The average soil temperature in the 0-40 cm soil layer at night in the I4 treatment was 0.4-0.9% lower than that in the I1 treatment (**Figures 3**, **4**).

At 55 DAE, the mean 5 cm soil temperature was 5.3-13.6% greater in the I1 treatment than in the I5 treatment (**Figures 3**, **4**). At 75 and 95 DAE, the soil temperatures of the 5 cm soil layer in the I5 treatment were 4.2-11.8% and 2.9-9.5% lower than those in the I1 treatment from 14:00-22:00, and the average temperatures from 0:00-10:00 were 0.4-1.0% and 0.6-2.6% lower, respectively. The soil temperatures in the 10 cm soil layer at 4:00-8:00 a.m. were 0.0-2.3% and 0.0-2.2% greater in the I4 treatment than in the I1 treatment at 55 and 75 DAE, respectively. At 55-95 DAE in 2020, the average soil temperature in the I1 treatment was 2.5-7.8% and 5.6-8.1% higher than that in the I4 and I5 treatments, respectively, in the 10 cm soil layer from 10:00-22:00. At 55-75 DAE, the I3, I4, and I5 treatments had 1.3-10.7%, 2.3-6.7%, and 1.9-7.6% lower soil temperatures, respectively, in the 20 cm soil layer from 12:00-22:00 than did the I1 treatment.

### Soil thermal conduction

**Figure 5:**
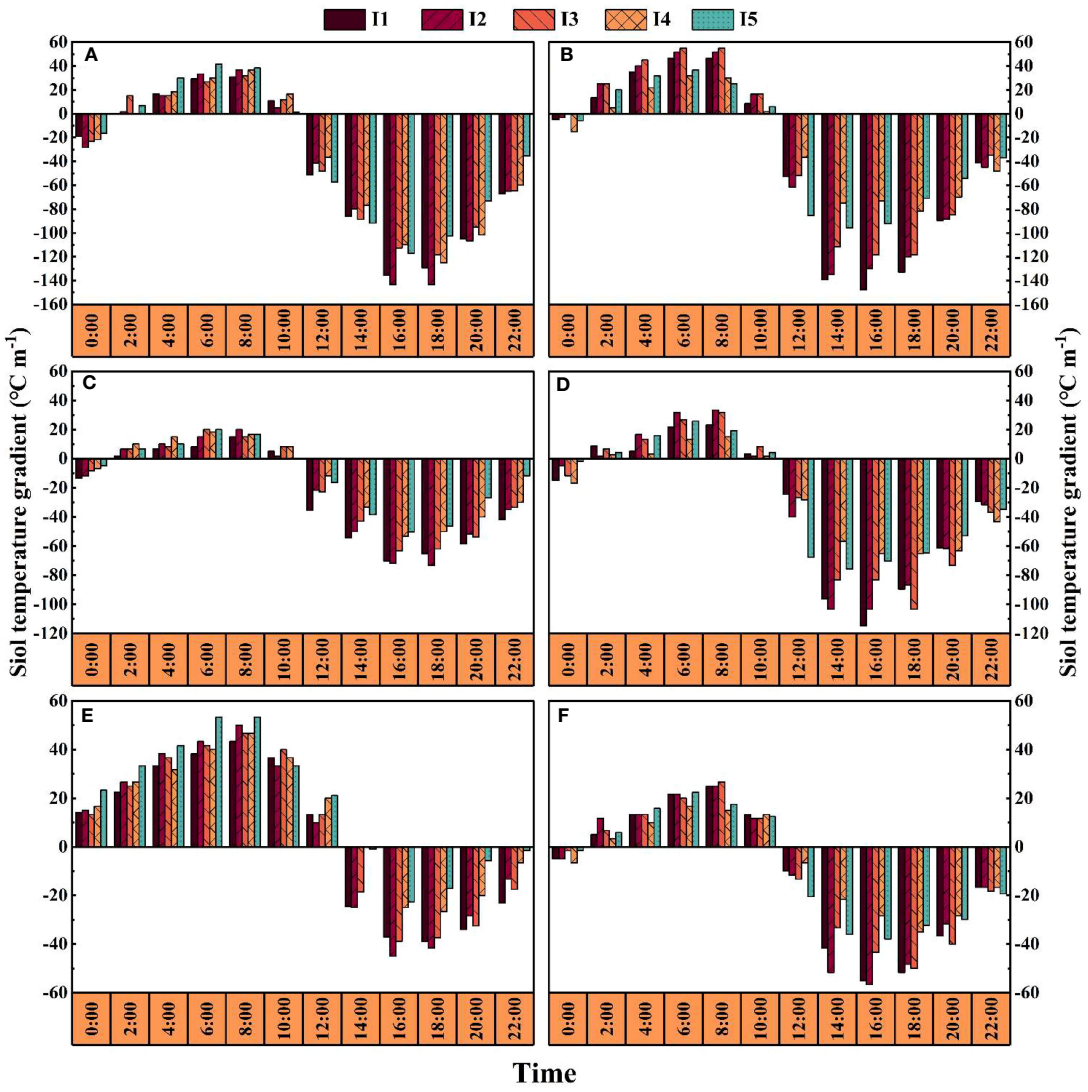
Effects of different irrigation amounts on the diurnal variation in thermal conduction. **(A, C, E)** represent 55, 75 and 95 days after emergence in the I1-I5 treatments in 2019, respectively; **(B, D, F)** represent 55, 75 and 95 days after emergence in the I1-I5 treatments in 2020, respectively.

The soil temperature gradient from 12:00-0:00 was mostly negative; from 14:00-18:00, the absolute value of the soil temperature gradient in each treatment reached the maximum, and the overall difference between the treatments was significantly greater in the I1 and I2 treatments than in the I4 and I5 treatments (**Figure 5**). In 2019–2020, the soil temperature gradients at 16:00 under the I1 treatment were 17.0–20.0%, 18.9–46.2%, and 13.8–31.2% and 9.7–13.4%, 24.0–41.1% and 21.5% greater than those in the I3, I4 and I5 treatments at 55 and 75 DAE, respectively. The soil temperature gradient at night (2:00-10:00) was mostly positive, with peak values from 6:00-8:00 and no significant trend between treatments.

### Leaf relative water content

**Figure 6:**
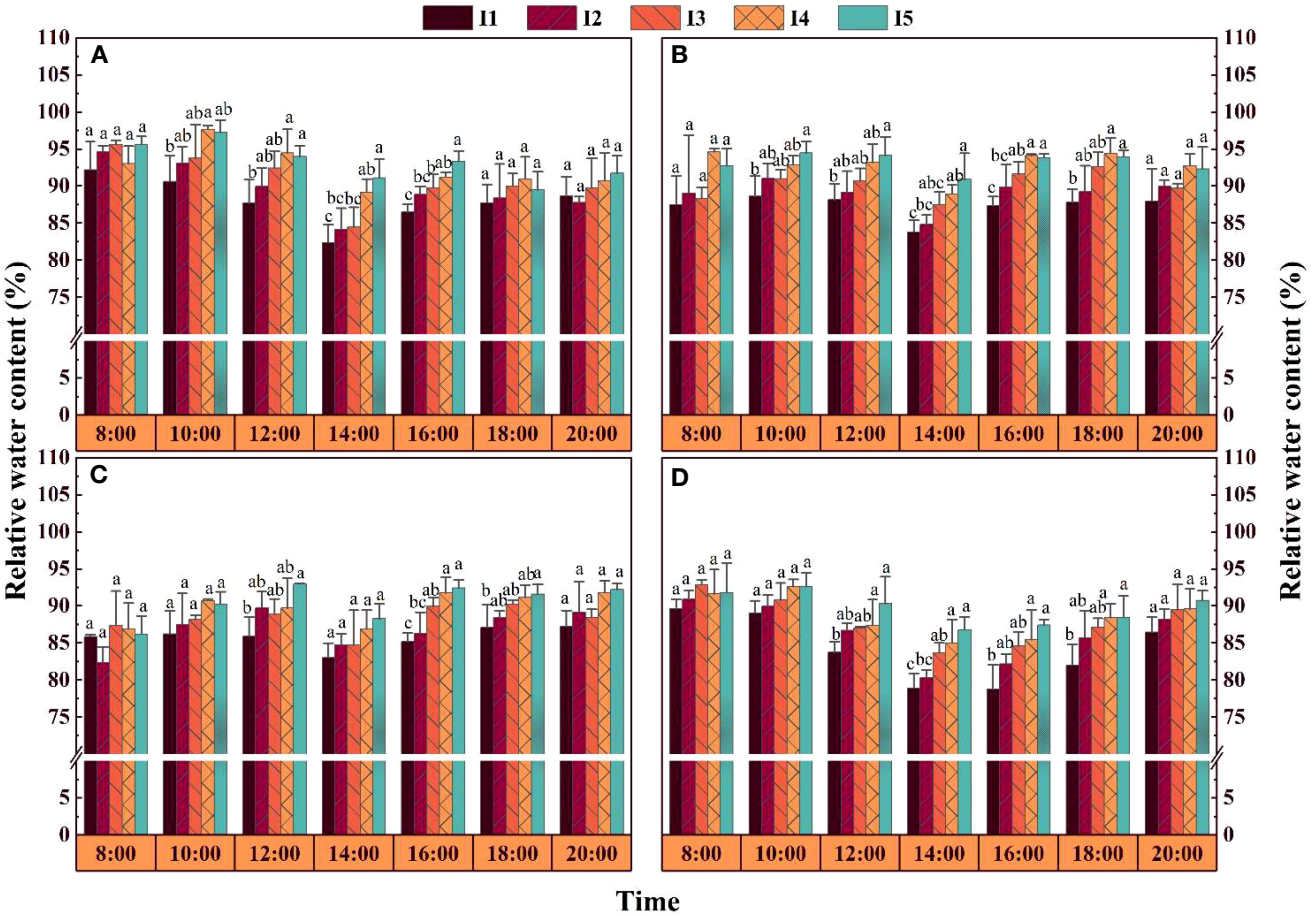
Effects of different irrigation amounts on the daily variation in the relative water content of leaves. **(A, B)** represent 70 and 90 days after emergence in the I1-I5 treatments in 2019, respectively; **(C, D)** represent 90 and 110 days after emergence in the I1-I5 treatments in 2020, respectively. The bars are the means of three repetitions, and the error bars represent the standard errors. Different letters above the adjacent five columns indicate statistical significance at the P = 0.05 level.

At 70, 90 (2019-2020 mean, same below), and 110 DAE, the daily average RWC in the I5 treatment was 3.9-5.6%, 4.3-5.8%, and 3.8-6.3% greater than that in the I1 and I2 treatments and 0.8-2.6%, 0.5-2.9%, and 1.3-2.0% greater than that in the I3 and I4 treatments, respectively (**Figure 6**). The RWC showed an overall decreasing trend from 8:00 to 14:00, reaching the lowest value at 14:00 and then gradually increasing. In the I1 treatment, the RWC at 12:00 decreased by 2.6-7.2%, 2.7-7.0%, and 3.4-7.2% compared with that in the other treatments at 70, 90, and 110 DAE, respectively, and there was a significant difference in RWC between the I1 and I5 treatments. At 70 DAE, the RWC at 14:00-16:00 in the I3 treatment decreased by 3.8-7.3% compared with that in the I5 treatment and by 1.6-5.3% compared with that in the I4 treatment, although the differences were not significant. At 90 DAE, the RWC in the I5 treatment from 16:00 to 18:00 increased by 4.7-6.9% compared with that in the I1 treatment in 2019 and by 4.9-7.8% compared with that in the I1 treatment in in 2020, with significant differences between the two treatments during this period.

### Leaf temperature

**Figure 7:**
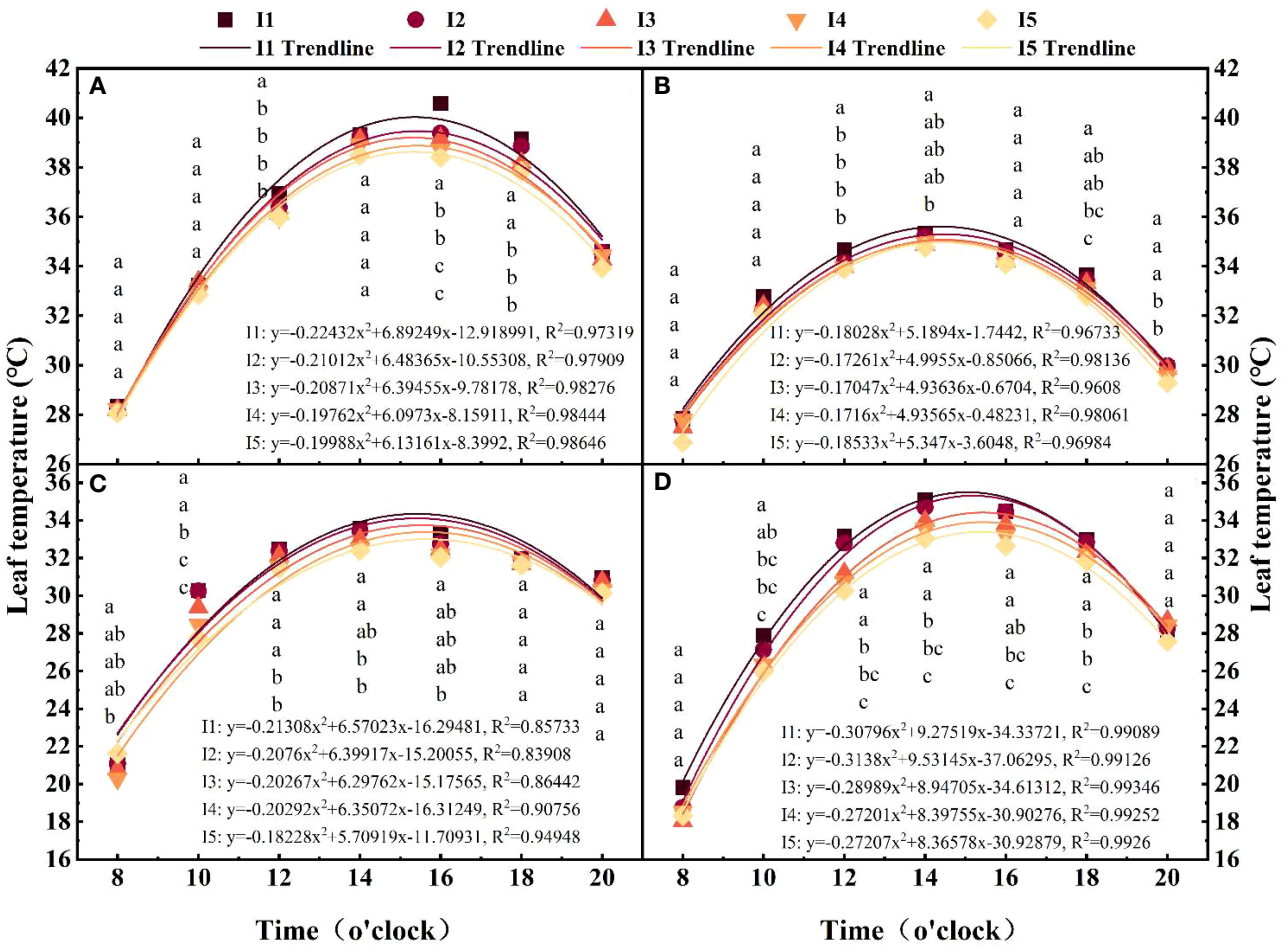
Effects of different irrigation amounts on daily changes in leaf temperature. **(A, B)** Leaf temperature changes in the I1-I5 treatments at 8:00-20:00 at 70 and 90 days after emergence in 2019; and **(C, D)** Leaf temperature changes in the I1-I5 treatments at 8:00-20:00 at 90 and 110 days after emergence in 2020. The points represent the means of three repeated measurements of the leaf temperature. Different letters indicate statistical significance at the P = 0.05 level.

With a decreasing irrigation amount, the cotton leaf temperature increased, and compared with those in the I4 and I5 treatments, the daily average temperature in the I1 treatment was 1.4-4.8% and 2.2-6.2% higher, respectively (**Figure 7**). The cotton leaf temperatures in the I3 and I4 treatments increased by 1.3-3.1% and 0.4-1.5% compared with that in the I5 treatment and decreased by 0.52.8% and 0.8-3.4% compared with that in the I2 treatment. The cotton leaf temperature showed an obvious temporal pattern with different growth stages. The leaf temperature followed a typical unimodal curve as the day progressed, first increasing and then decreasing, with a peak from 14:00 to 16:00, conforming to the binomial growth model. The R^2^ reached 0.83908-0.99089. At 12:00, the leaf temperature in the I1 treatment was significantly greater than that in the I4-I5 treatments, with increases of 2.7%, 2.5-2.7% and 7.2-8.7% at 70, 90 and 110 DAE, respectively. At 16:00, the leaf temperature of the I4 treatment group was 1.7%, 1.4%, and 3.7% and 1.1%, 0.6%, and 1.8% lower than those of the I2 and I3 treatments at 70, 90 and 110 DAE, respectively.

### Gas exchange parameters

**Figure 8:**
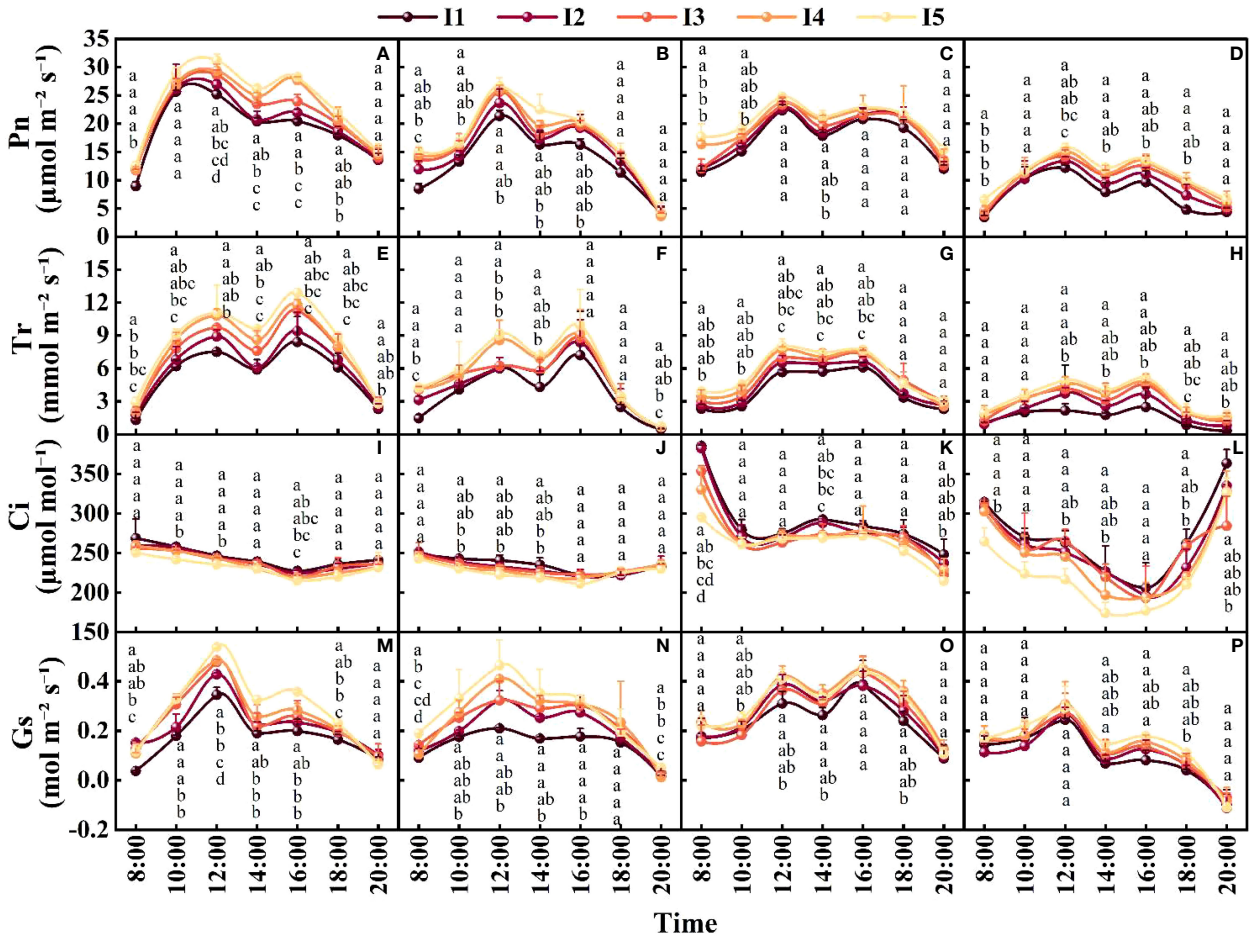
Effects of different irrigation amounts on gas exchange parameters in 2019 and 2020. **(A, E, I, M)** represent the changes in the Pn, Tr, Ci, and Gs of cotton leaves 70 days after emergence in 2019; **(B, F, J, N)** represent the changes in the Pn, Tr, Ci, and Gs of cotton leaves 90 days after emergence in 2019. **(C, G, K, O)** represent the changes in the Pn, Tr, Ci, and Gs of cotton leaves 90 days after emergence in 2020; and **(D, H, L, P)** represent the changes in the Pn, Tr, Ci, and Gs of cotton leaves 110 days after emergence in 2020. The points represent the means of three repetitions, and the error bars represent the standard errors. Different letters indicate statistical significance at the P = 0.05 level.

At 70, 90, and 110 DAE, the daily average Pn in the I4 treatment was 4.8-15.3%, 4.8-16.8%, and 5.9-26.3% higher, respectively, than those in the I1-3 treatments and 5.0%, 4.7%, and 6.8% lower, respectively, than that in the I5 treatment (**Figures 8A–D**). As the day advanced, the cotton leaf Pn exhibited an “M-shaped” bimodal curve; the peak periods occurred at 12:00 and 16:00, and the peak value was lower at 16:00 than at 12:00. At 12:00 at 70, 90, and 110 DAE, the average Pn in the I5 treatment increased by 5.9-19.3%, 3.1-14.8%, and 4.1-22.2% compared with those in the other treatments.

With an increase in the irrigation amount, the Tr increased, and the average daily Tr at 70, 90 and 110 DAE was 8.1-34.2%, 6.3-32.3% and 8.8-55.2% higher, respectively, in the I5 treatment than in the I1-I4 treatments (**Figures 8E–H**). As the day progressed, Tr exhibited a bimodal pattern of change, with peaks occurring at 12:00 and 16:00. At 70, 90, and 110 DAE, there were periodic valleys in the Tr value in each treatment at 14:00. Compared to those in the I1 treatment, the values in the I5 treatment were 38.4%, 31.8%, and 56.0% greater, respectively, and the differences were significant. There was no significant difference compared to the I4 treatment, and the results were similar between years.

As the irrigation amount increased, the Ci decreased. The daily average Ci of cotton was 1.6-5.3%, 1.7-7.1%, and 5.1-16.5% greater in the I1 treatment than in the I2-5 treatments at 70, 90, and 110 DAE, respectively (**Figures 8I–L**). As the day progressed, Ci showed a “V”-shaped trend (except at 90 DAE in 2020), with the lowest value occurring at 16:00. At this time, compared with that at 8:00, the Ci in the I3-I4 treatments decreased by 14.1-15.4% and 35.9-38.4% at 70 DAE and 110 DAE, respectively, and there was no significant difference between these two treatments.

The daily average Gs increased with increasing irrigation amount (**Figures 8M–P**). Compared with those in the other treatments, the percentages in the I5 treatment at 70, 90, and 110 DAE increased by 11.4-38.7%, 4.8-34.5%, and 7.8-35.8%, respectively. As the day progressed, the Gs followed an “M-shaped” bimodal variation curve, with peak periods occurring at 12:00 and 16:00. At 70 and 90 DAE, the value in the I5 treatment increased by 35.9% and 39.7%, respectively, compared to that in the I1 treatment, and the difference was significant; in the I4 treatment, these values increased by 9.9% and 5.0%, respectively, without significant differences. Compared with the values at 14:00, those recorded at 16:00 increased by 4.4-15.3%, 8.1-18.4% and 16.3-30.7% at 70, 90 and 110 DAE, respectively, in the I1-I5 treatments.

### Water use efficiency and lint yield

The WUE in the I3 treatment decreased by 15.8%, 16.8%, and 32.9% compared with that in the I1 treatment and increased by 7.2%, 3.6%, and 15.8% compared with that in the I5 treatment at 70, 90, and 110 DAE, respectively (**Table 3**). Additionally, the WUE in the I4 treatment decreased by 17.4%, 24.1%, and 42.7% compared with that in the I1 treatment and increased by 5.4%, 0.3%, and 1.3% compared with that in the I5 treatment at 70, 90, and 110 DAE, respectively. At 90 DAE, the WUE in the I3 treatment increased by 18.2% compared with that in the I5 treatment at 12:00, and there was no significant difference compared to that in the I4 treatment. At 16:00 on 70 and 110 DAE, the values in the I3 treatment were 18.8% and 46.1% lower than those in the I1 treatment and 9.8% and 0.7% greater than those in the I4 treatment, respectively. There was no significant difference between the I3 and I4 treatments. The lint yield increased with increasing irrigation amount, and overall, the trend was I3, I4>I5>I1, and I2. In 2019–2020, in comparison with the I1, I2, and I5 treatments, the I4 treatment significantly increased by 12.2–18.0%, 8.7–18.7%, and 7.0–12.1%, respectively. Compared with the I3 treatment, the I4 treatment increased by 4.4-7.4%, with no significant difference.

**Table 3:** Effect of irrigation amount on water use efficiency and lint yield without mulching.

Year		Days after emergence (d)	Time	I1	I2	I3	I4	I5
2019	WUE (μmol mmol^-1^)	70	8:00	7.10 ± 1.52 a	6.75 ± 0.77 ab	5.92 ± 1.20 ab	5.87 ± 0.48 ab	4.50 ± 0.74 b
			10:00	4.16 ± 0.40 a	4.07 ± 1.32 a	3.49 ± 0.53 a	3.18 ± 0.26 a	3.21 ± 0.20 a
			12:00	3.35 ± 0.09 a	3.03 ± 0.18 a	2.94 ± 0.15 a	2.74 ± 0.26 a	2.95 ± 0.64 a
			14:00	3.54 ± 0.70 a	3.39 ± 0.22 a	3.08 ± 0.15 a	2.94 ± 0.36 a	2.72 ± 0.08 a
			16:00	2.61 ± 0.22 a	2.39 ± 0.33 ab	2.12 ± 0.10b	2.35 ± 0.15 ab	2.19 ± 0.03 ab
			18:00	3.08 ± 0.58 a	2.73 ± 0.13 a	2.60 ± 0.40 a	2.57 ± 0.29 a	2.46 ± 0.09 a
			20:00	5.99 ± 0.69 a	5.67 ± 1.00 a	4.97 ± 0.62 a	4.99 ± 0.39 a	5.29 ± 0.31 a
		90	8:00	6.04 ± 0.75 a	3.92 ± 0.94 b	3.38 ± 0.23 b	3.51 ± 0.16 b	3.57 ± 0.08 b
			10:00	3.27 ± 0.32 a	3.14 ± 0.24 a	2.96 ± 0.41 a	3.06 ± 0.33 a	3.18 ± 0.93 a
			12:00	3.56 ± 0.16 ab	3.96 ± 0.71 ab	4.11 ± 0.18 a	3.17 ± 0.62 ab	2.94 ± 0.22 b
			14:00	4.03 ± 0.97 a	3.01 ± 0.26 a	3.23 ± 0.46 a	2.85 ± 0.13 a	3.12 ± 0.36 a
			16:00	2.96 ± 1.29 a	2.4 ± 0.48 a	2.23 ± 0.11 a	2.34 ± 0.42 a	2.33 ± 0.79 a
			18:00	4.52 ± 0.83 a	4.52 ± 1.70 a	5.19 ± 1.86 a	4.46 ± 0.45 a	4.23 ± 0.54 a
			20:00	8.04 ± 1.19 a	5.94 ± 1.30 a	5.24 ± 0.57 a	5.23 ± 1.15 a	5.86 ± 2.00 a
	Lint yield (kg ha^-1^)			1214 ± 64 c	1204 ± 113 c	1392 ± 34 ab	1480 ± 53 a	1300 ± 61 bc
2020	WUE (μmol mmol^-1^)	90	8:00	5.04 ± 0.87 a	4.54 ± 0.02 a	4.6 ± 0.26 a	4.73 ± 0.25 a	4.73 ± 0.25 a
			10:00	6.21 ± 1.20 a	5.61 ± 0.58 a	5.4 ± 1.17 a	4.47 ± 0.33 a	4.47 ± 0.33 a
			12:00	3.95 ± 0.03 a	3.53 ± 0.54 ab	3.35 ± 0.04 ab	3.09 ± 0.16 b	3.09 ± 0.16 b
			14:00	3.14 ± 0.32 a	2.87 ± 0.10 a	2.87 ± 0.22 a	2.98 ± 0.24 a	2.98 ± 0.24 a
			16:00	3.45 ± 0.33 a	3.28 ± 0.16 a	2.94 ± 0.11 a	3.06 ± 0.55 a	3.06 ± 0.55 a
			18:00	5.75 ± 0.13 a	5.67 ± 0.44 ab	4.76 ± 0.57 ab	4.57 ± 0.37 ab	4.57 ± 0.37 ab
			20:00	5.78 ± 0.82 a	4.57 ± 0.41 a	4.49 ± 0.39 a	5.49 ± 0.25 a	5.49 ± 0.25 a
		110	8:00	3.66 ± 0.64 a	4.23 ± 1.12 a	3.16 ± 0.47 a	2.85 ± 0.65 a	3.53 ± 0.86 a
			10:00	5.27 ± 1.44 a	4.4 ± 0.20 a	3.9 ± 1.40 a	3.76 ± 1.44 a	3.32 ± 0.63 a
			12:00	6.21 ± 0.81 a	3.67 ± 0.59 b	3.46 ± 0.49 b	3.42 ± 0.77 b	3.45 ± 0.31 b
			14:00	4.49 ± 1.02 a	3.7 ± 0.48 a	4.53 ± 1.15 a	2.98 ± 0.31 a	3.06 ± 0.35 a
			16:00	5.14 ± 0.67 a	3.22 ± 1.05 b	2.77 ± 0.53 b	2.79 ± 0.13 b	2.64 ± 0.08 b
			18:00	5.59 ± 0.58 a	5.45 ± 1.54 a	4.65 ± 0.73 a	5.17 ± 1.40 a	5.06 ± 1.54 a
			20:00	13.73 ± 1.94 a	10.38 ± 5.46 ab	7.13 ± 2.63 ab	4.28 ± 1.51 b	3.85 ± 0.79 b
	Lint yield (kg ha^-1^)			1832 ± 43 c	1905 ± 18 bc	1994 ± 10 ab	2086 ± 80 a	1939 ± 55 bc

Values are means ± SEs. Within a row in the same year, values followed by different letters are significantly different (P< 0.05) according to Duncan’s multiple range test.

### Correlation analysis

**Figure 9:**
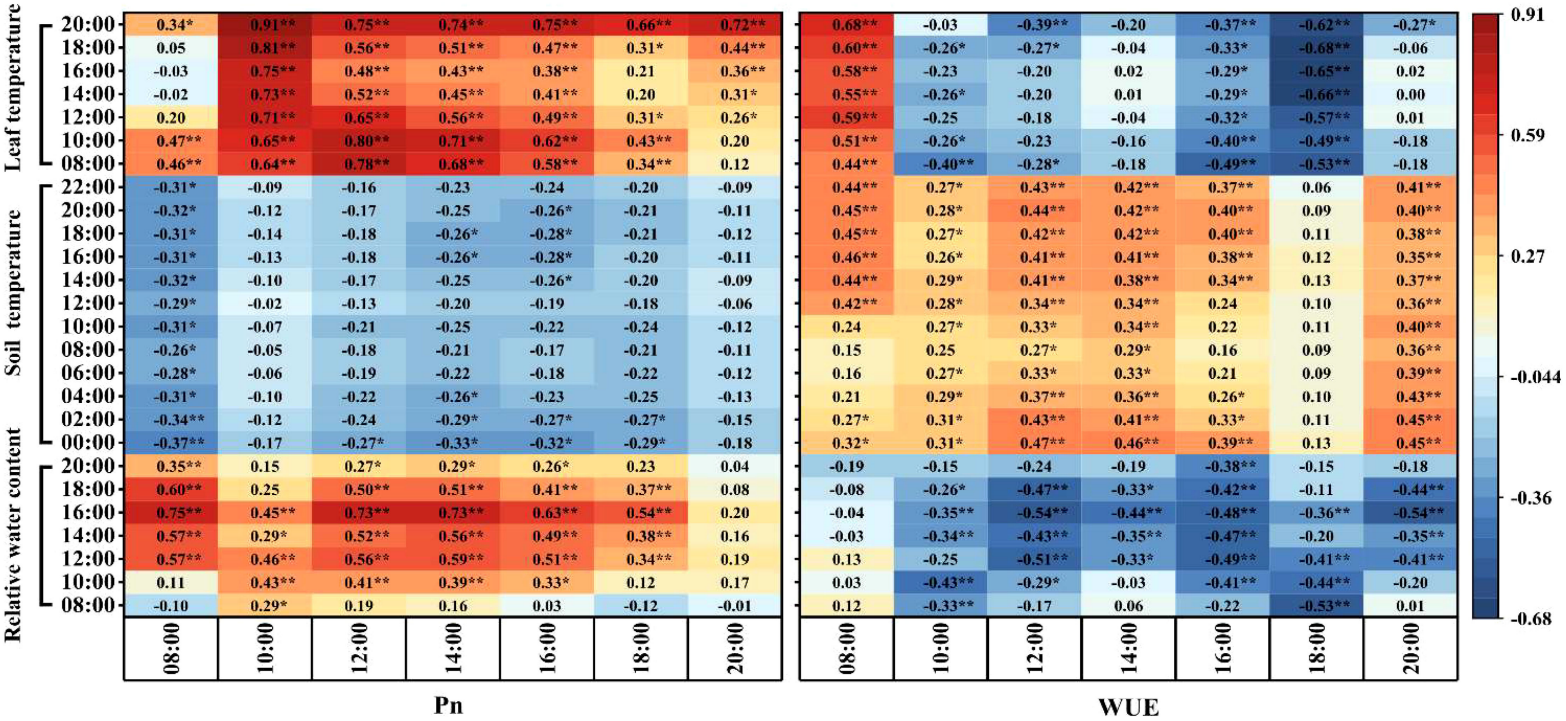
Correlations between soil temperature, leaf temperature, and leaf relative water content and Pn and WUE. * and ** represent significance levels of 5% and 1%, respectively.

Correlation analysis revealed that the RWC of leaves from 10:00 to 18:00 was significantly or extremely significantly and positively correlated with Pn from 10:00 to 16:00 and was significantly or extremely significantly and negatively correlated with WUE from 12:00 to 16:00 (**Figure 9**). The all-day soil temperature was positively correlated with WUE from 10:00 to 16:00 and at 20:00, while leaf temperature was highly significantly and positively correlated with Pn from 10:00 to 16:00 and significantly or highly significantly and negatively correlated with WUE from 16:00 to 18:00. The results indicated that the leaf hydrothermal environment mainly positively affected the leaf net photosynthetic rate from 10:00 to 16:00, and the soil temperature environment mainly positively affected the WUE from 10:00 to 16:00.

## Discussion

### Moderately reducing the irrigation amount can optimize the distribution of soil moisture content and improve the soil temperature in nighttime subsurface irrigation systems without mulching

Variations in soil moisture content are crucial in influencing crop growth and yield (Guan et al., 2013). Research on surface drip irrigation without mulching has revealed pronounced fluctuations in soil moisture levels within the 0-20 cm layer under various irrigation conditions (Wang et al., 2020). Compared with the drip irrigation without mulching treatment, the I3-I5 treatments with nighttime subsurface irrigation without mulching increased the moisture content of the 20-60 cm soil layer, while the moisture content of the 0-20 cm soil layer was less affected than that under conventional drip irrigation conditions without mulching (**Figure 2**). This indicated that nighttime subsurface irrigation without mulching can reduce the moisture content in the surface soil and promote water transfer, thus reducing the evaporation of water caused by the lack of film cover.

The soil temperature gradient is a physical characteristic that measures the vertical direction and intensity of heat transfer in the soil (Füllner et al., 2011; Ao et al., 2014). Research has shown that the temperature gradient in the soil at night is positive, reaching a peak in each treatment from 6:00 to 8:00 (**Figure 5**), indicating that the temperature in the upper soil decreases after heat dissipates into the atmosphere at night, heat is transferred from the deep soil to the surface soil, and there is a great hysteresis phenomenon in the transfer of the soil temperature (Huo et al., 2019). In addition, this study found that reducing irrigation can greatly increase soil temperature (**Figures 3**, **4**), but it is not conducive to weakening the amplitude of the diurnal variation in soil temperature (Li et al., 2013; Jiao et al., 2014; Zhao et al., 2014). However, while maintaining a high temperature is conducive to the early growth of cotton, it may also inhibit root and microbial activities in the middle and late growth periods and accelerate plant senescence (Funaba et al., 2006). Therefore, appropriately reducing the amount of irrigation can optimize the distribution of soil moisture in different soil layers to increase the soil temperature, which is conducive to growing cotton without mulching.

### Moderately reducing the irrigation amount can lead to the maintenance of a greater Tr and Pn when cotton enters the photosynthetic ‘lunch break’

Plants respond to water stress by closing their stomata and reducing transpiration loss, thereby increasing leaf temperature (Monteiro et al., 2016). Studies have shown that water stress reduces the Pn, Gs, Tr, and Ci of cotton leaves (Li et al., 2004). However, we found that with decreasing irrigation amount, the Pn and Gs in the leaves decrease, while the Ci increases (**Figure 8**). This may be the result of nonstomatal factors (Frederick and Camberato, 1994). In addition, researchers have suggested that reducing irrigation can reduce the leaf area of cotton plants, leading to a decrease in leaf temperature (Shahenshah and Isoda, 2015). Our results revealed that with decreasing irrigation, the diurnal leaf Tr and RWC decrease simultaneously (**Figures 6**, **8**), while the leaf temperature increases (**Figure 7**). This may be because cotton leaves consume some energy through their own transpiration, and when water conditions are insufficient, transpiration decreases, the energy consumed decreases, and the blade temperature increases accordingly.

Photosynthetic lunch breaks are beneficial ecological adaptations and self-regulatory mechanisms for hot conditions and are controlled by ecological and physiological factors (Yokoyama et al., 2019). In this study, due to high light availability and temperature stress at midday, the leaf Pn under the I1 treatment was significantly lower than that under the I5 treatment, the peak value occurred earlier, and the degree of photosynthetic rest was more severe (**Figure 8**). However, after a period of high temperature and light availability, the photosynthetic function of leaves gradually recovered, and there was no significant difference in the net photosynthetic rate between treatments at 20:00 (Zhang et al., 2002). At 14:00, the Tr of the I5 treatment was significantly greater than that of the I1 treatment, and there was no significant difference from that of the I3-I4 treatments, indicating that the treatment with a greater irrigation amount had a greater soil water supply capacity throughout the day, which was an important reason for the greater stomatal conductance, photosynthetic intensity and lint yield (**Figure 8**).

### Moderately reducing the irrigation amount can improve the coordination between yield and WUE and eliminate residual film pollution

Different irrigation amounts can lead to varying accumulations and redistributions of photosynthetic assimilates, thereby affecting yield and WUE (Chen et al., 2019; Gao et al., 2021). In this study, the I4 treatment increased the lint yield by 1.1% compared to the overall average lint yield in Xinjiang, and the I3 and I4 treatments increased the lint yield compared with that of the I5 treatment (**Table 3**). This indicated that in the nighttime subsurface irrigation system without mulching, the cotton yield can reach the average yield obtained in the local mulch-covered cotton field (National Bureau of Statistics of China, 2021) while saving 276-552 m^3^ ha^-1^ of water. In addition, researchers have suggested that increasing soil temperature can increase the stomatal conductance of crop leaves, thereby increasing the CO_2_ concentration in the leaves and promoting leaf photosynthesis (Huang et al., 2021). Through correlation analysis, our results revealed that there is a negative correlation between daytime and nighttime soil temperature and Pn (**Figure 9**), indicating that excessive soil temperature can inhibit photosynthesis, leading to the loss of photosynthetic products and a decrease in yield. However, excessively low soil temperature is not conducive to root water absorption or nutrient acquisition, resulting in delayed crop growth and development (Jia et al., 2019). In addition, we found that leaf temperature and RWC are positively correlated with the overall Pn, indicating that increasing leaf temperature and leaf RWC is conducive to increasing the photosynthetic capacity of cotton, which agrees with studies showing that nighttime warming can promote the postanthesis photosynthetic capacity of wheat (Fan et al., 2015). In summary, in the subsurface nighttime irrigation system without mulching, the I3-I4 treatments improved the distribution of the soil moisture content, enhanced the soil temperature, and maintained high RWC, Gs, Tr, and Pn values during the day, thereby increasing the lint yield and reducing irrigation water consumption; additionally, the I3-I4 treatments have the potential to replace cotton under film drip irrigation systems in arid areas and similar areas worldwide.

The most direct and effective way to increase yield is to increase planting density (Chen et al., 2019). Due to uncontrollable factors such as weather, the cotton seedling density in 2020 was greater than that in 2019, which may be the primary reason for the overall yield increase in 2020. Therefore, in order to evaluate the sustainability of the recommended system, further evaluation of the relationship between seedling density and the efficient utilization of water and heat resources in farmlands is needed.

## Conclusion

**Figure 10:**
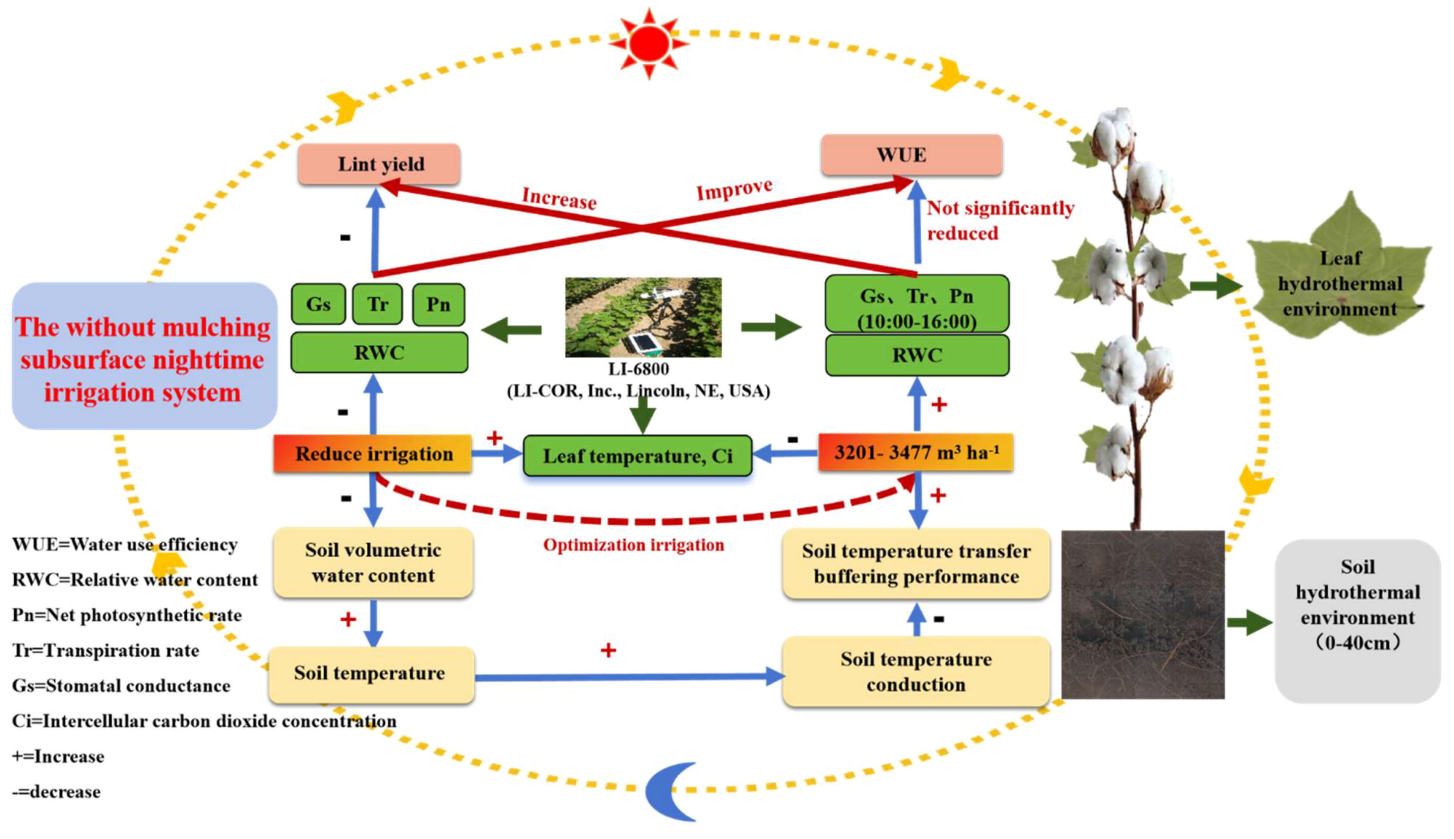
A working model to achieve stable lint yield and water use efficiency by optimizing the irrigation amount in a nighttime subsurface irrigation system without mulching.

An appropriate reduction in the irrigation amount (I3 and I4 treatments) increases the soil temperature and thermal conductivity by reducing the soil moisture content in the 0-40 cm soil layer. It also increases the leaf temperature, Ci, and maintains high RWC, Pn, Gs and Tr, thereby increasing yield (**Figure 10**). There was no significant difference in WUE among the I3, I4, and I5 treatments from 8:00 to 20:00. Correlation analysis shows that the hydrothermal environment of the leaves had a positive impact on the Pn, and the soil temperature had a positive impact on WUE during the same period (10:00-16:00). Overall, in the nighttime subsurface irrigation system without mulching, an irrigation amount of 3201-3477 m^3^ ha^-1^ is conducive to creating a suitable soil and leaf hydrothermal environment; maintaining a high photosynthetic capacity from 10:00 to 16:00; increasing yield; and stabilizing the WUE. This system may replace mulched drip irrigation systems for cotton cultivation, thus reducing the harmful effects of residual film pollution while saving irrigation water.

## Data availability statement

The raw data supporting the conclusions of this article will be made available by the authors, without undue reservation.

## Author contributions

N-nL: Writing – original draft, Conceptualization. J-hL: Writing – review & editing, Conceptualization. X-jS: Writing – review & editing, Investigation. FS: Writing – review & editing, Investigation. YT: Writing – review & editing, Investigation. JW: Writing – review & editing. X-zH: Writing – review & editing, Investigation. Z-bW: Writing – review & editing. H-hL: Writing – review & editing.
